# A combination of 5-azacytidine and nivolumab is a potentially effective rescue therapy in relapsed/refractory AITL

**DOI:** 10.3389/fimmu.2024.1410638

**Published:** 2024-06-25

**Authors:** Laure Ricard, Pascale Cervera, Nicolas Stocker, Elise Corre, Zoé Van de Wyngaert, Anne Banet, Zora Marjanovic, Rémy Dulery, Clotilde Bravetti, Anne-Christine Joly, Minh Tam Baylatry, Paul Coppo

**Affiliations:** ^1^ Sorbonne Université, Institut National de la Santé Et de la Recherche Médicale (INSERM), Centre de Recherche Saint-Antoine (CRSA), Paris, France; ^2^ Service d’Hématologie, Hôpital Saint-Antoine, Assistance Publique - Hôpitaux de Paris (AP-HP)- Sorbonne Université, Paris, France; ^3^ Service d’Anatomopathologie, Assistance Publique - Hôpitaux de Paris (AP-HP) - Sorbonne Université, Paris, France; ^4^ Centre de Référence des Microangiopathies Thrombotiques (CNR-MAT), Hôpital Saint-Antoine, Assistance Publique - Hôpitaux de Paris (AP-HP) - Sorbonne Université, Paris, France; ^5^ Service d’hématologie biologique, Assistance Publique - Hôpitaux de Paris (AP-HP) - Sorbonne Université, Paris, France; ^6^ Hôpital Saint-Antoine, Pharmacie, Assistance Publique - Hôpitaux de Paris (AP-HP) - Sorbonne Université, Paris, France

**Keywords:** angioimmunoblastic T cell lymphoma, 5-azacytidine, nivolumab, T follicular helper cell, TET2, RhoA

## Abstract

**Introduction:**

Angioimmunoblastic T-cell lymphoma (AITL) is a peripheral T-cell lymphoma characterized by a T follicular helper cell phenotype expressing PD-1 (programmed cell death-1). AITL exhibits a poor response to conventional chemotherapy, with a median 5-year overall survival of 44% and a progression-free survival of 32%. Relapse is common, resulting in a median overall survival of 6 months. Recurrent mutations are detected in genes regulating DNA methylation, including TET2, DNMT3A, and IDH2 variants, along with the prevalent RHOA G17V mutation. In this context, patients treated with the hypomethylating agent 5-azacytidine achieved overall response and complete response rates of 75% and 41%, respectively. We hypothesized that targeted therapies combining anti-PD-1 checkpoint blockers with hypomethylating agents could be efficient in AITL patients and less toxic than standard chemotherapy.

**Methods:**

Here, we report the efficacy of a regimen combining 5-azacytidine and nivolumab in nine relapsed or refractory AITL patients.

**Results:**

This regimen was well-tolerated, especially in elderly patients. The overall response rate was 78%, including four partial responses (44%) and three complete responses (33%). Allogeneic hematopoietic stem cell transplantation was performed in two patients who reached complete response.

**Discussion:**

These preliminary favorable results may serve as a basis for further investigation in prospective studies.

## Introduction

Angioimmunoblastic T-cell lymphoma (AITL) is a subtype of peripheral T-cell lymphoma (PTCL) characterized by a T follicular helper cell phenotype. AITL predominantly affects older individuals, with a median age of 65 years (1). The prognosis is bleak, with a 44% median 5-year overall survival and an 32% progression-free survival (PFS) rate. One of the standard first-line therapeutic approach remains Cyclophosphamide-Doxorubicin-Vincristine-Prednisone (CHOP) therapy. Unfortunately, relapse is frequent, with a median post relapse overall survival of only 6 months (2).

AITL originates from CD4+ T follicular helper cells (Tfh) and is characterized by an exacerbated inflammatory response and immune dysregulation. Molecular studies have identified pathogenic variants in genes regulating DNA methylation and a dysregulation in T-cell signaling. Specifically, *TET2*, *DNMT3A*, and *IDH2* variants are present in 80%, 25%, and 25% of patients with Tfh-derived PTCL, respectively (3, 4). Notably, these mutations alone are insufficient for lymphomagenesis, and 70% of AITL patients also carry a recurrent *RHOA* G17V mutation. Mouse models support the notion that a combination of *RHOA* G17V mutations with *TET2* mutations is necessary to induce lymphomagenesis with a Tfh phenotype (5). First initiator mutations involve epigenetic regulators (TET2 or DNMT3A); secondly, driver-mutations such as RHOA G17V and IDH2 R172K/S promote the expansion of clonal Tfh cells (6).

CD4+ Tfh cells are crucial for germinal center T- and B-cell development, and express the checkpoint inhibitor PD-1 (programmed cell death-1). In AITL lymph nodes, malignant Tfh cells represent a minority of cellular components, coexisting with various immune cells such as immunoblasts, eosinophils, and plasma cells. Most immunoblasts are Epstein-Barr virus (EBV)-infected (1), contributing to increased PDL-1 expression and creating a tolerogenic milieu favoring malignant cell survival while suppressing neighboring macrophages and effector T cells (7, 8). PD-1 expression is detected in 80% of AITL cases, correlating with a poor prognosis (9). Of note, the highly recurrent activating mutation (p.Gly17Val) in the RhoA small GTPase promotes CD4+ T cell polarization in Tfh cells with expression of CXCR5 and PD-1 (10).

Building upon these findings, targeted therapies combining anti-PD-1 checkpoint blockers with hypomethylating agents may offer enhanced efficiency in AITL treatment, while potentially reducing toxicity compared to standard chemotherapy. A retrospective study of 12 AITL patients treated with the single hypomethylating agent 5-azacytidine reported an overall response rate of 75% and a complete response rate of 41% (11). Furthermore, a phase 1 study demonstrated the efficacy of a combination of romidepsin with 5-azacytidine in eight of 11 PTCL patients, including three with complete responses in AITL cases (12) A phase 2 study confirmed the efficacy of 5-azacitidine and romidepsin in 25 treatment-naïve PTCL patients, particularly in those with a Tfh phenotype (13). A phase 3 study comparing 5-azacytidine to romidepsin, gemcitabine, or bendamustine in relapsed or refractory AITL patients showed a median PFS of 5.6 months (95%CI, 2.66–8.11) in the 5-azacytidine arm versus 2.8 months in the standard treatment group (95%CI, 1.87–4.83). In the ORACLE trial, 5-azacytidine exhibited a better safety profile and an overall response rate of 33%, with 11% of patients achieving a complete response (14). A phase 1 study reported an overall response rate of 40% using the PD-1 checkpoint blocker nivolumab in five refractory or relapsed PTCL patients (15). A phase 2 study reported modest activity of the single agent tislelizumab, a programmed cell death protein 1 inhibitor, in 44 patients with refractory PTCL including 11 AITL patients with an ORR of 20.5% and a CR rate of 9.1% (16). We provide here our experience of a therapy combining 5-azacytidine and nivolumab (5-Aza/Nivo) through a compassionate use in nine relapsed or refractory AITL patients when no other therapeutic options were available.

## Methods

In this monocentric and retrospective study, patients received 5-azacytidine at 75 mg/m^2^ subcutaneously for 7 consecutive days every 28 days and nivolumab at a dose of 3mg/kg every 14 days (5-Aza/Nivo) until progression or until achieving a complete response before undergoing allogeneic hematopoietic stem cell transplantation (allo-HSCT), if deemed eligible. Response to treatment was assessed clinically and using positron emission tomography-computed tomography scans (PET-scanner) every two cycles, according to standardized recommendations (17). Expert pathologists from the national program “Lymphopath” confirmed the AITL diagnosis based on the World Health Organization 2016 classification (18). DNA sequencing was performed using deep next-generation sequencing (NGS) with a 47-gene capture panel (**Supplementary Method**).

This study was conducted in compliance with the Good Clinical Practice protocol and the principles of the Declaration of Helsinki.

## Results

Nine patients underwent treatment with the 5-Aza/Nivo regimen (**Table 1**). The median age was 69 (interquartile range [IQR] 56–82). None of the patients had a concurrent myelodysplastic/myeloproliferative neoplasm. All individuals presented with advanced disease (stage III-IV), with six patients exhibiting cutaneous lesions and one patient having bone marrow involvement. Two patients had a poor performance status (PS>2), and the median international prognostic index (IPI) was 3 at the time of diagnosis. Eight patients had elevated C-reactive protein (CRP), and five patients had elevated ß2-microglobulin. One patient experienced hypercalcemia >3.0 mmol/L, one had severe autoimmune hemolytic anemia and thrombocytopenia, and another had hypereosinophilia. All patients had relapsed or refractory AITL following a median of one (IQR 1–2) therapies before initiating the 5-Aza/Nivo regimen. First-line therapies included CHOP (n=6), reduced dose CHOP (mini-CHOP) (n=2), or CHOEP (n=1). The median time before relapse/progression counted from start/end of treatment was 6.1 months (IQR 0.8–14.2). Three patients received a second-line therapy consisting of brentuximab vedotin, ifosfamide, carboplatin, and etoposide (BrICE) (19). The median time before the second relapse was 2.8 months (IQR 2.7–6.2).

**Table 1:** Patient’s characteristics at baseline.

ID	Age at diagnosis	Sex	IPI	stage	LDH>ULN	ECOG	Extranodal sites	B2M>ULN	CRP>ULN	B symptoms	NGS Gene (VAF%)	Status at last Follow up	Number of previous therapy	Number of 5-Aza/Nivo cycles	Best response	Relapse/progression	Allo-HSCT	PFS (months)
AITL1	82	M	3	3	no	2	1	NA	yes	yes	*TET2* p.Ser385*,(13%) *TET2* p.Leu532*, (11%)	Dead	1	4	PR	yes	no	4
AITL2	62	F	3	4	yes	1	1	yes	no	yes	*DNMT3*(31%) *RHOA* (6%) *TET2* Exon 03 c.3341del (8%) *TET 2Exon 04 c.3467del* (32%)	Alive	2	5	CR	no	yes	>24
AITL3	81	M	4	3	no	2	1	yes	yes	yes	*TP53 (84%)* *DNMT3 (74%)* *TET2 (50%)*	Dead	2	7	PR	yes	no	7
AITL 4	56	M	2	4	yes	1	1	NA	yes	no	No mutation	Dead	2	6	PR	yes	no	6
AITL 5	69	M	3	3	yes	3	0	NA	yes	yes	*TET2* Exon 11 c.4636C>T (11%) *TET2* Exon 3 c.3320C>G (11%) *PLCG1* (2%) *RHOA* (2%) *IDH2* (2%)	Dead	1	3	CR	no	yes	15
AITL 6	56	M	3	4	yes	2	1	yes	yes	yes	*TET2 Exon 3* *c.2255_2261delATAAAGA (*8%) *TET2 Exon3 c.2428C>T (*8%) *PLCG1 (*6%) *IDH2 (*6%*)*	Dead	1	4	PR	yes	no	3
AITL 7	71	F	4	3	yes	3	1	yes	yes	yes	*DNMT3A (40%)* *TET 2 Exon 3 c.2739delA (39%)* *TET2 Exon 10 c.4184T>A (37%)* *CD28 (7%)* *RHOA (5%)*	Dead	1	1	PD	yes	no	0
AITL 8	52	M	2	4	yes		2	yes	yes	yes	No NGS analysis	Alive	1	4	CR	yes	no	3
AITL 9	74	M	2	3	no	1	0	NA	yes	yes	*TET2 (12%) RHOA (3%)*	Alive	1	4	PD	yes	no	2

M, male; F, female; IPI, international prognostic index; LDH, lactate dehydrogenase; ECOG/PS, Eastern Cooperative Oncology Group/Performance status; B2M, ß2-microglobulin; NA, not applicable; NGS, next generation sequencing; VAF, variant allele frequency; CRP, C-reactive protein; ULN, upper limit of normal value; PR, partial response; CR, complete response, PD, progression; PFS, progression-free survival.

**Figure 1:**
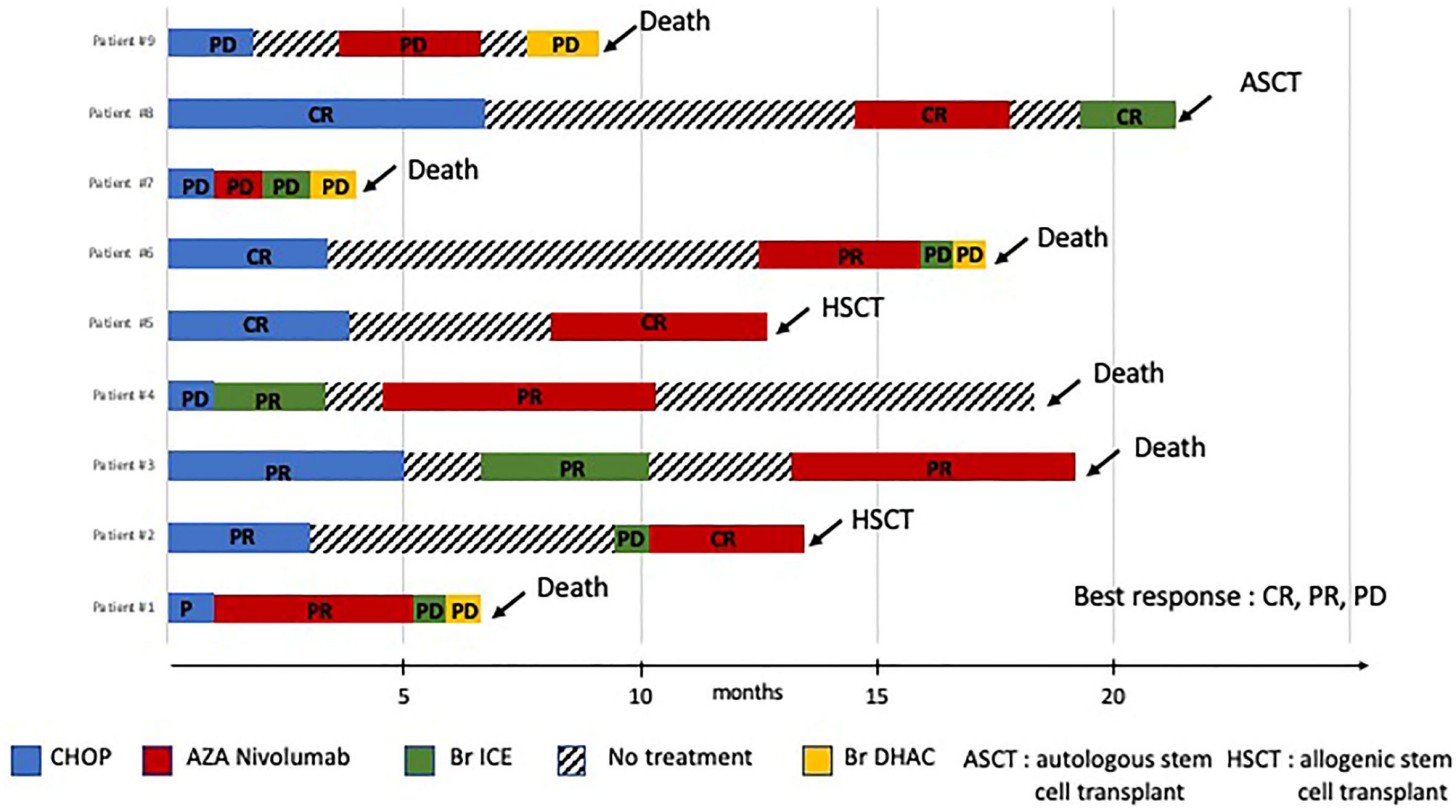
Time course of patients from first-line treatment to latest follow-up. The best response and duration of response during the different treatments are shown on the plots. CR, complete response, PR, partial response, PD, progressive disease. HSCT, hematopoietic stem cell transplantation.

**Figure 2:**
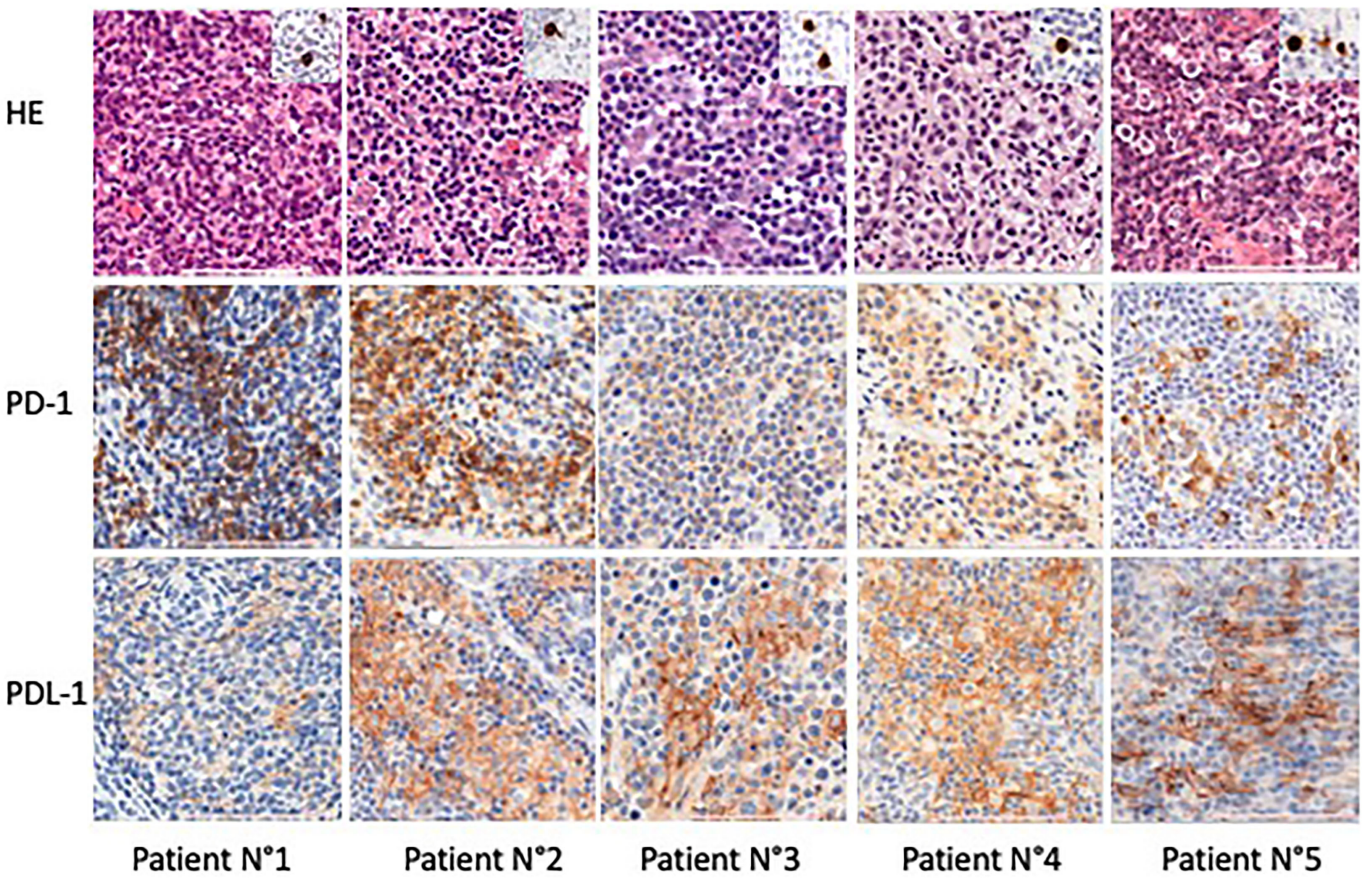
Examples of PD-1 and PD-L1 expression in angioimmunoblastic T-cell lymphoma in some patients. x40 Hematoxylin-eosin staining showing typical histopathological features of angioimmunoblastic T-cell lymphoma. Immunostaining with PD-1 and PD-L1 highlighting tumoral T cells. Immunostaining is performed with anti-PD-1 (prediluted, clone NAT105; Roche) and anti-PDL-1 (1/100, Clone QR1; Diagomics) antibodies. The Leica Bond III automated platform (LEICA, Nanterre, France), is used, according to the protocols included in the instructions for antibodies use, applying Bond epitope retrieval solution 1 and 2, respectively for each antibody, 1 (10mn) and 2 (20mn). Tonsil tissue is included on each slide, as positive control.

Treatment with the 5-Aza/Nivo regimen took place between May 2020 and February 2023. Patients received a median of 4 courses (IQR 1–7) (**Figure 1**). Rituximab was added for two patients due to EBV-induced B-cell proliferation associated with polyarthralgia in one and steroid-refractory autoimmune hemolytic anemia with thrombocytopenia in the other. Lymph node biopsies revealed EBV-positive immunoblasts in all patients, along with circulating EBV DNA (median PCR 4.2 log IQR (3,95–4,56)). Tfh tumor cells expressed PD-1 and PDL-1, indicating immune tolerance against tumor proliferation and potential immune checkpoint inhibitors response markers (**Figure 2**). NGS was performed in 8 patients; seven harbored *TET2* mutations alone (1 case) or in association with *DNMT3A* (3 cases), or with *RHOA* mutations (4 cases), or with *IDH2* mutations (1 case), *TP53* (1 cases), and *CD28* mutation (1 case). In one patient, no mutation was found, possibly due to too few tumor cells (**Table 1**).

The tolerance of the treatment was acceptable. All patients experienced grade 1 to 4 adverse events (AEs), but these were mostly transient and readily manageable. Most grade >2 events were sepsis and cytopenias, especially anemia. Infectious events were a picc-related *Klebsiella pneumoniae* bacteremia, a grade 2 prostatitis and a *Clostridium difficile* infection (one case each), all of favorable outcome. One patient experienced a colitis with grade 4 diarrhea related to anti-PD-1 treatment, which responded favorably to corticosteroids while nivolumab was continued. One additional patient had hypothyroidism with no detectable anti-thyroid antibodies. No treatment-related deaths were reported (details in **Table 2**).

**Table 2:** Patients’ complications on vidaza nivolumab regimen.

	Infection	Cytopenia	Other drug-related events
Patient #1	2 grade 3 Picc-related bloodstream infections (*Staphylococus epidermidis*, *Klebsiella pneumoniae*)	Anemia grade 1	no
Patient #2	no	no	Fever related to Nivolumab infusion grade 1
Patient #3	Prostatitis grade 2	Anemia grade 1	Colitis grade 4
Patient #4	*Clostridium difficile* colitis grade 2	Anemia grade 1	no
Patient #5	no	no	Nausea grade 2
Patient #6	no	Neutropenia grade 4	no
Patient #7	no	Anemia grade 2	no
Patient #8	no	no	Hypothyroidism grade 2
Patient #9	no	Anemia grade 3	no

The overall response rate was 78%, including four partial responses (44%) and three complete responses (33%). Notably, one patient with progressive disease after CHOP and BrICE regimens achieved a complete response after 5 courses of 5-azacytidine and 4 courses of nivolumab, followed by an allo-HSCT (**Figure 1**). The patient remains in persistent complete remission 24 months after allo-HSCT. Another patient with progressive disease following six courses of CHOP achieved a complete response after two courses of 5-Aza/Nivo; subsequently, an allo-HSCT was performed. This patient was still in complete remission but succumbed to COVID-19 pneumonia 9 months after allo-HSCT. One patient refractory to CHOEP, achieved complete remission after two 5-Aza/Nivo courses but experienced progression after the fourth cycle, precluding the planned allo-HSCT. He was then treated with BrICE (Br ICE, 3 cycles) and an autologous stem cell transplantation was performed. The 5-Aza/Nivo regimen was discontinued in the remaining 6 patients due to progressive disease, after a progression-free survival of 3 months (IQR 0.7–7). Six patients died 0 to 7 months later from progressive disease. Nivolumab can induce autoimmune adverse events. None of the patients developed autoimmune complications, including the patient who presented with autoimmune cytopenia at diagnosis. We did not detect a correlation between response to treatment and the number of *TET2* mutations, or the presence of *RHOA, DNMT3A* or *IDH2* mutations.

## Discussion

We present here the first AITL patients treated with a regimen combining 5-azacytidine and nivolumab after standard treatment failed to control the disease. We hypothesized here that nivolumab and azacytidine could act synergistically to confer more response opportunities in these patients with a dismal prognosis. In line with this view, the majority of patients responded to our regimen, with three cases of complete response. Tolerance was acceptable, supporting the feasibility of this combination, even in elderly and frail patients. Interestingly, this combination could represent an acceptable bridging therapy in patients suitable for an allo-HSCT. A prospective phase 2 study analyzed the effect of single agent nivolumab in refractory peripheral T-cell lymphoma. The study was held because of short-duration response and cases of hyper-progression, particularly in AITL patients (20). Interestingly, we did not observe any case of hyper-progression here, possibly thanks to the combined use of 5-azacytidine. Adding PDL-1 blockers to other conventional agents showed benefit in non-Hodgkin B cell lymphoma compared to the limited response when given as a single agent (21). In that regard, PDL-1 blockers and PD-1 blockade have been reported to be effective in patients with an aggressive NK/T-cell lymphoma at relapse, as tumor cells harboring EBV genome upregulate PDL-1 (22).

We observed here an encouraging overall response rate of 75% with our regimen. Other doublet regimens for R/R PTCL have also allowed achieving achieved responses in 60% to 80% of patients. Like in our report, the majority of these studies were small phase 1 or phase 2 studies including highly selected patients (12, 13, 23, 24). As opposed to other studies, we included frail patients over 80 years old. Although our results cannot formally demonstrate that nivolumab and 5-azacytidine act synergistically in patients with AITL, we provide evidence that this combination therapy seems at least reasonable in R/R AITL with acceptable side effects. Similarly to other studies, because of the small number of patients and the limited number of patients with wild type *TET2*, we could not provide a correlation between *TET2* mutations and response to 5-azacytidine with anti PDL-1 therapy; interestingly however, two of the three patients who achieved a complete response harbored two *TET2* mutations with *RHOA* mutation (11, 13). Although stemming from a limited number of cases, this observation further supports the rationale of a strategy combining a differentiating agent targeting epigenetic alterations with a checkpoint inhibitor in AITL patients.

Taken together, our preliminary results support 5-azacytidine and nivolumab as a potentially effective rescue combination in relapsed/refractory AITL, and the need for further evaluation of this regimen through formal clinical trials.

## Data availability statement

The original contributions presented in the study are included in the article/**Supplementary Material**. Further inquiries can be directed to the corresponding author.

## Ethics statement

Ethical approval was obtained as necessary from local ethical committee. The studies were conducted in accordance with the local legislation and institutional requirements. Written informed consent for participation was not required from the participants or the participants’ legal guardians/next of kin in accordance with the national legislation and institutional requirements.

## Author contributions

LR: Writing – original draft, Writing – review & editing. PCe: Writing – review & editing. NS: Writing – review & editing. EC: Writing – review & editing. ZV: Writing – review & editing. AB: Writing – review & editing. ZM: Writing – review & editing. RD: Writing – review & editing. CB: Formal analysis, Writing – original draft. A-CJ: Writing – review & editing. MB: Writing – review & editing. PCo: Writing – original draft.
