# Supplementary material for: A combination of 5-azacytidine and nivolumab is a potentially effective rescue therapy in relapsed/refractory AITL
Source: Front Immunol. 2024 Jun 25;15:1410638. doi: 10.3389/fimmu.2024.1410638 (PMC11231067; doi:10.3389/fimmu.2024.1410638)
Supplement: Supplementary file 1 [file Table_1.docx]

**Supplemental method**

We performed targeted sequencing of 47 genes associated with B and/or T lymphomas (*ARID1A*, *ATM*, *BCL2*, *BIRC3* (exons 7 to 10), *BRAF* (exon 15), *BTK*, *CARD11* (exon 4 to 10), *CCDN1* (exon 1), *CD28*, *CD37*, *CD79A* (exon 4 to 5), *CD79B*, *CXCR4* (exon 2), *DMT3A, EGR2*, *EZH2* (exon 16 to 17), *FBWX7*, *ID3*, *IDH2* (exon 4), *IGLL5, JAK3, KLF2*, *KRAS*, *MYC*, *MYD88* (exon 3 to 5), *NFKBIE*, *NOTCH1* (exon 34 and 3’UTR), *NOTCH2* (exon 34 and 3’UTR), *PIM1*, *PLCG1*, *PLCG2* , *POT1*, *PTPRD*, *RHOA*, *RPS15*, *SAMHD1*, *SF3B1* (exon 12 to 18), *SMARCA4*, *SPI1* (exon 5), *STAT3* (exon 19 to 12), *STAT5B* (exon 14 to 17), *STAT6* (exon 12 to 18), *TCF3*, *TET2*, *TNFAIP3*, *TP53*, *XPO1* (exon 15), using custom capture kit (Sure Select Custom, Agilent Technologies®). After enzymatic fragmentation of genomic DNA, libraries were generated using the Sure Select SureSelect XT Low Input Kit (Agilent Technologies®) according to the manufacturer’s protocol and paired-end sequencing (2 x 150 bp reads) was performed on an MiSeq (Illumina®) sequencer. Sequence reads were aligned to the reference genome GRCh37 and analysed using Sequence Pilot software (JSI medical systems®). Variants were identified with a VAF cutoff 2% and a minimum of 20 reads covering the variant. The mean depth within the targeted regions was 900X.
